# Acute appendicitis as an unusual cause of invasive ductal breast carcinoma metastasis

**DOI:** 10.1093/jscr/rjaa535

**Published:** 2020-12-28

**Authors:** Vincent De Pauw, Julie Navez, Stephane Holbrechts, Jean Lemaitre

**Affiliations:** Department of Abdominal Surgery, CHU Ambroise-Paré, Mons, Belgium; Medico-Surgical Department of Gastroenterology, Hepatopancreatology and Digestive Oncology, Erasme Hospital, Université Libre de Bruxelles, Brussels, Belgium; Medico-Surgical Department of Gastroenterology, Hepatopancreatology and Digestive Oncology, Erasme Hospital, Université Libre de Bruxelles, Brussels, Belgium; Department of Abdominal Surgery, CHU Ambroise-Paré, Mons, Belgium; Department of Abdominal Surgery, CHU Ambroise-Paré, Mons, Belgium

## Abstract

Acute appendicitis is one of the most common causes of abdominal pain at the emergency room. In rare cases, it can be caused by malignancy, even metastatic lesions from extra-abdominal neoplasia. Herein, we report a case of a 64-year-old female with a history of invasive ductal carcinoma of the breast treated by chemotherapy, surgery, radiotherapy and hormonotherapy, relapsing several years later as a bone and a pleura metastasis successfully cured by locoregional therapy and hormonal treatment. She presented with acute abdominal pain without signs of peritonitis. Abdominal computed tomodensitometry showed sign of appendicitis. Therefore, laparoscopic exploration and appendicectomy was performed. During surgery, multiple peritoneal nodules were found and harvested. Pathology showed metastatic nodules of invasive ductal breast carcinoma, including in the appendicular wall, concluding to peritoneal carcinomatosis. The postoperative course was uneventful, but the patient died 1 year later after refusing anticancer treatment.

## INTRODUCTION

Breast cancer (BC) is the most frequently diagnosed cancer in women, representing 11.6% of all new cancer cases in 2018. Its prognosis significantly improved during the last three decades, thanks to better screening and medico-surgical treatments. However, around 10% of patients will develop metastasis, most frequently in bones, liver, lungs or brain. Metastasis to gastrointestinal tract and peritoneum are uncommon, especially to appendix for which clinical presentation and imaging can mimic appendicitis. Acute appendicitis is one of the most common causes of abdominal pain at the emergency department. Usually due to obstruction of the lumen at the appendicular basis (by stercolith or lymphoid hyperplasia), a malignant tumor is found at final pathology in <1%. We present the case of a 64-year-old female who was treated for an acute appendicitis, which eventually turned out to be a metachronous metastases from BC developed 20 years before. This case report was approved by the Institutional Review Board of our institution and was performed in accordance with the precept of the Declaration of Helsinki.

## CASE REPORT

A 64-year-old woman was admitted to the emergency department for hypogastric and right lower abdominal quadrant pain that appeared 15 days ago. The pain presented as transient crisis lasting about 10–20 min, without any triggering factor and without analgesic position. The patient mentioned a weight loss of 8 kg within the last 2 months.

In her past medical history, she had a breast ductal carcinoma 20 years ago, treated by neoadjuvant chemotherapy, right mastectomy, adjuvant radiotherapy and hormonal therapy (tamoxifen) for 5 years. She relapsed 10 years later as a sternal metastasis, treated by radiotherapy and a 6-year treatment with exemestane and bisphosphonate. Shortly after stopping hormonal treatment, a right pleural metastatic lesion was diagnosed on computed tomodensitometry (CT), not responding to taxol, and treated by thoracoscopic talcage 4 years ago. The disease stabilized with an ongoing letrozole-based treatment.

**Figure 1:**
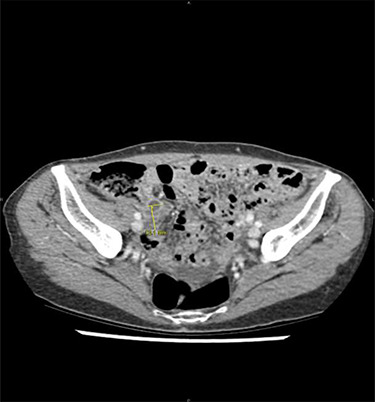
Acute appendicitis on abdominal computed tomodensitometry (yellow arrow), characterized hereby by a dilatation of the appendicular lumen (12 mm) with wall thickening.

At clinical examination, abdominal pain was localized at the McBurney area, without signs of peritonitis; intestinal transit was preserved. Laboratory results showed a C-reactive protein at 2.1 mg/dl (*N*<1), white blood cells at 15 000 μl/m^3^ (*N*<10 000) and no liver or renal function tests alteration. On abdominal CT, a dilated appendix (12 mm) was observed with wall thickening and periappendiceal fluid, suggesting acute appendicitis (Fig. 1). An emergency appendicectomy was therefore indicated. At laparoscopic exploration, an inflamed appendix was observed as well as multiple and unexplained nodules, including an indurated lesion on the appendix. Appendicectomy was performed by ligation of the appendicular basis, and some peritoneal nodules were harvested. The postoperative course was uneventful.

Pathological analysis identified a 75 mm long appendix with massive infiltration of the appendix by a metastasis of poorly differentiated ductal carcinoma from mammary glands, identical to peritoneal nodules. Tumoral cells had a glandular aspect, with clear and intense positivity of progesterone receptors at immunostaining, a weaker positivity of estrogen receptors (ER) and membrane positivity for E-cadherin; there was no staining for Human Epidermal Growth Factor Receptor-2 (HER-2). The proliferative activity was estimated between 5 and 10%.

**Figure 2:**
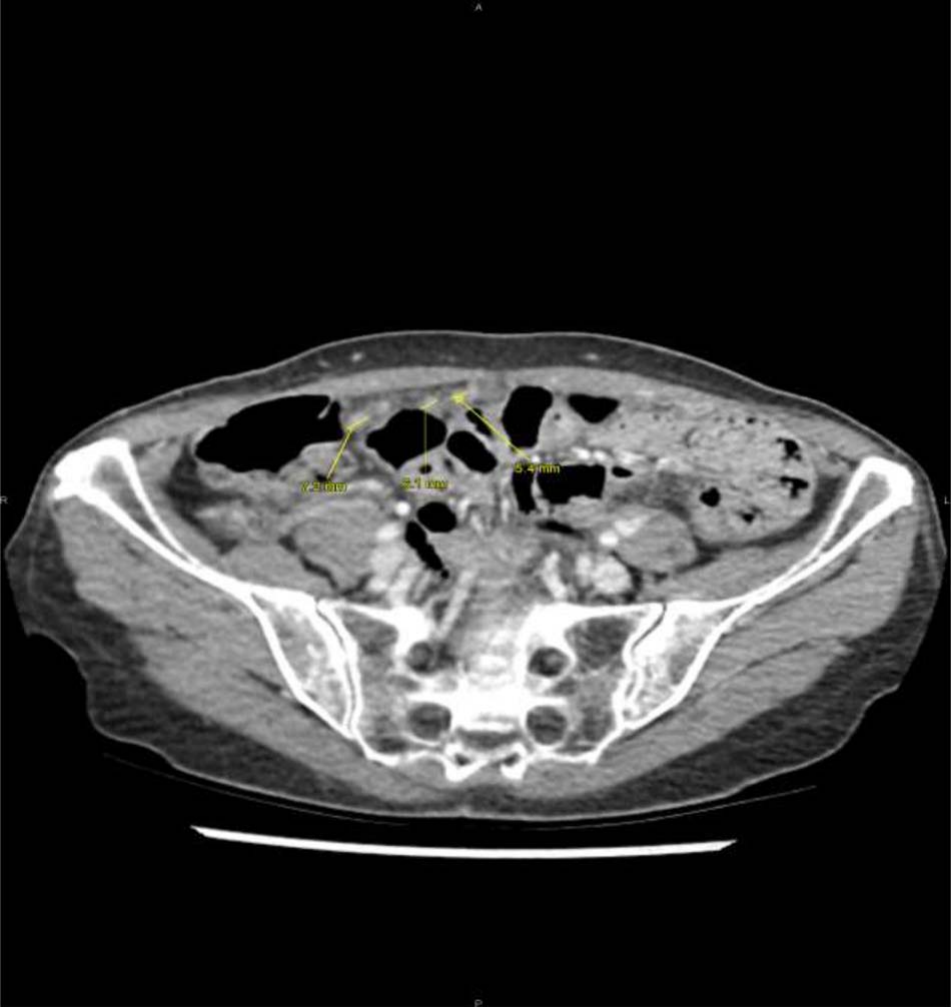
Peritoneal noduli observed after retrospective analysis of abdominal computed tomodensitometry (yellow arrow).

After refusing any anticancer treatment therapy, the patient died 1 year after appendicectomy. By retrospectively analysing imaging performed at the emergency department, we observe that the nodules of peritoneal carcinomatosis could be identifiable on CT images (Fig. 2).

## DISCUSSION

Approximately 10% of patients with BC develop secondary lesions, either synchronous (20%) or metachronous (80%) after a median follow-up of 7 years [1]. Peritoneal carcinomatosis is relatively rare, with an estimated prevalence of 0.7% of BC cases. Predictive factors for developing peritoneal carcinomatosis are high grading tumor, lobular invasive histological subtype and advanced TNM stage. Once peritoneal metastasis appeared, the prognosis of BC become very poor [2]. Surgical cytoreduction and hyperthermic intraperitoneal chemotherapy showed encouraging results in selected patients reported in small retrospective studies, but remains difficult to evaluate because of the low prevalence of peritoneal carcinomatosis from BC [2, 3].

Clinical presentation of peritoneal carcinomatosis from BC is unspecific, variable and poorly described [4–6]. Because of its rarity, no specific abdominal imaging is dedicated in the routine follow-up after treatment of BC, except liver ultrasonography for detection of liver metastases. In the present case, an acute appendicitis was diagnosed without other abdominal lesions, but after retrospective analysis of CT imaging, peritoneal nodules could be identified. The radiological diagnosis of peritoneal carcinomatosis is often challenging, especially without suspecting cancer recurrence. This highlights the role of the radiologist in diagnosing based on symptoms and medical past history, and not only on imaging. Although there is scarcity of such situation, all patients with abdominal symptoms and a history of BC should be considered at risk of peritoneal metastasis. Anyway, the present patient would have been managed in the same way, requiring appendicectomy for treating the acute episode and for making the histological diagnosis of BC recurrence.

Regarding histological subtype, lobular carcinoma, representing 10% of BC, is more likely to metastasize to abdominal organs and peritoneum than ductal carcinoma [2, 7]. This may be related to biomolecular features. Positive E-cadherin expression is frequently associated with invasive lobular carcinoma and has been cited as a possible risk factor of distant metastasis [8]. Overexpression of HER-2 receptor has been associated with a fast tumor growth and a poorer prognosis, and ER-negative BC has also a higher propensity to metastasize to abdominal viscera [9, 10]. The present case is quite exceptional because of the uncommon disease evolution for a ductal breast carcinoma that recurred in the peritoneal cavity, with positive E-cadherin expression, absence of HER-2 staining and ER-positivity, which made the patient at lower risk of peritoneal and gastrointestinal recurrence of her BC.

In conclusion, acute appendicitis caused by metastasis from invasive ductal breast carcinoma is rare. Although the occurrence of peritoneal carcinomatosis in BC is low, one should be aware of this diagnosis, especially in patients with a history of mammary cancer even after a long period of recurrence. This can be manifested by any kind of abdominal symptoms, including acute appendicitis, and abdominal imaging is mandatory and specifically analysed with the clinical neoplastic context.
